# Measles and Rubella Immunity Profile of Healthcare Workers in Bulgaria: A Cross-Sectional Study

**DOI:** 10.3390/vaccines14090727

**Published:** 2026-08-23

**Authors:** Stefka Krumova, Zhivka Getsova, Lora Veleva, Radostina Stefanova, Tencho Tenev, Elitsa Golkocheva-Markova

**Affiliations:** 1 National Reference Laboratory of Measles, Mumps, Rubella, Department of Virology, National Center of Infectious and Parasitic Diseases, 1504 Sofia, Bulgaria; loraveleva9@gmail.com (L.V.); rss_94@abv.bg (R.S.); 2 Center of Competence “ImmunoPathogen”, 1000 Sofia, Bulgaria; getsova@ncipd.org (Z.G.); tdtenev60@gmail.com (T.T.); elmarkova2007@gmail.com (E.G.-M.); 3 Department of Epidemiology, National Center of Infectious and Parasitic Diseases, 1504 Sofia, Bulgaria; 4 National Reference Laboratory of Viral Hepatitis, Department of Virology, National Center of Infectious and Parasitic Diseases, 1504 Sofia, Bulgaria

**Keywords:** measles, rubella, protective immunity, IgG, healthcare

## Abstract

Healthcare workers (HCWs) and medical providers are regularly exposed to a wide range of infectious diseases in the course of their duties. This occupational risk is significant in the context of highly contagious infections such as measles, which can spread rapidly in healthcare institutions. This study aimed to assess the seroprevalence of protective anti-measles and anti-rubella IgG antibodies and factors influencing immunity among HCWs across all 28 districts in Bulgaria. Methods: A total of 1854 individuals, including 540 (29%) doctors and 1314 (71%) medical specialists from all 28 districts in Bulgaria, were screened for protective immunity against measles and rubella. Serum samples were analyzed for the presence of protective IgG antibodies by ELISA (Enzyme-Linked Immunosorbent Assay) using commercial EUROIMMUN Anti-Measles/Anti-Rubella Virus IgG ELISA kits. According to the manufacturer’s instructions, the results were interpreted semi-quantitatively as positive, negative, and equivocal. Results: Anti-measles protective IgG antibodies were detected in 86.1% of participants for measles and in 95.2% for rubella. Our study found a statistically significant difference when analyzing demographic factors (age) for measles: the samples from older participants (over 50-year-old persons) were characterized by higher immunity levels; staff working in non-infectious-disease wards (*n*+ = 95.7%) were more frequently positive for rubella IgG antibodies compared to staff employed in infectious wards; and region locations varied for both groups. Gender and professional roles were not significant predictors. Conclusions: HCWs are at a greater risk of contracting infections than the general public because they have contact with sick patients or infectious material. Their vaccination or IgG immunity status must be strictly monitored, especially to limit the spread of nosocomial infections in hospital settings.

## 1. Introduction

Vaccine-preventable diseases (VPDs), especially measles and rubella, continue to be significant public health challenges worldwide, despite the existence of safe and highly effective vaccines for many years [1]. Measles, caused by a highly contagious morbillivirus, can result in severe complications such as pneumonia, encephalitis, and even death, particularly in vulnerable populations [2]. While rubella generally presents as a mild self-limiting illness in adults, it poses a serious risk to pregnant women due to the potential for Congenital Rubella Syndrome (CRS), which can cause severe fetal malformations or miscarriage [3]. In recent years, vaccination coverage has fallen short of optimal levels, compounded by increased vaccine hesitancy and pandemic-related disruptions to routine immunization programs. This has contributed to a resurgence of measles and rubella outbreaks worldwide [4]. According to the World Health Organization (WHO), certain groups face a significantly higher risk of infection and severe complications, namely non-immune persons (not vaccinated or vaccinated but without developed immunity), children who are not yet of optimal immunization age, immunocompromised individuals, and pregnant women.

In the healthcare setting, the transmission of pathogens poses a significant risk to infection control. Healthcare workers (HCWs) are at a much greater risk of occupational exposure due to their direct interaction with infected patients [5]. The nosocomial transmission of measles and rubella is a well-established issue that presents a dual threat: non-immune HCWs not only face the risk of contracting these infections themselves but can also serve as important carriers, spreading the viruses to vulnerable patients who are unvaccinated or immunocompromised [6]. Therefore, ensuring strong and verifiable immunity among the healthcare workforce is essential for both patient safety and the health of the workers [7].

Bulgaria has implemented measles vaccination for more than five decades; however, changes in vaccine formulations and schedules over time have created distinct immunity profiles among different birth cohorts of healthcare workers. The monovalent measles vaccine was introduced into the national immunization programme in the late 1960s, followed by universal childhood vaccination in the early 1970s. Initially, a single-dose strategy was applied, while a routine two-dose measles vaccination schedule was introduced in 1983. The combined measles–mumps–rubella (MMR) vaccine was incorporated into the national immunization programme in the early 1990s, initially as a first dose in infancy with a later second dose, and since 2001 a routine two-dose MMR schedule has been implemented [8].

Similar transitions from monovalent measles vaccination to combined MMR schedules occurred across Europe, although the timing differed between countries. Many Western European countries introduced MMR during the 1980s–1990s, followed by adoption of routine two-dose schedules to improve population immunity and prevent outbreaks. For example, two-dose MMR schedules became standard in many European countries during the 1990s, although differences remain in the timing of introduction, target ages, catch-up campaigns, and adolescent vaccination strategies. These variations contribute to heterogeneous immunity patterns among adult populations, including healthcare workers, who may belong to different vaccine-era cohorts [9].

Current HCWs represent several immunization cohorts: older personnel may have acquired immunity through natural measles infection or early vaccination programmes, whereas younger HCWs were generally vaccinated within the two-dose MMR era. Nevertheless, incomplete vaccination histories, missed second doses, differences in vaccine uptake between birth cohorts, and possible waning of detectable IgG antibodies may contribute to the persistence of susceptible individuals among HCWs. Therefore, the observed measles immunity profile among healthcare personnel reflects not only current vaccination coverage but also the cumulative impact of historical changes in national immunization policy. These cohort-related differences highlight the challenges of assessing measles and rubella immunity among HCWs solely based on age or vaccination history and underline the importance of reliable methods for confirming individual immune status.

Although many countries have immunization guidelines that recommend or require measles and rubella vaccinations for HCWs, reliance solely on historical vaccination records or self-reported vaccination history may be insufficient [10]. Factors such as waning vaccine-induced immunity over time, incomplete or missing documentation, and variations in historical national immunization schedules can lead to significant discrepancies between recorded vaccinations and actual protective serological immunity [11]. Serological screening, which involves measuring protective immunoglobulin G (IgG) antibodies, is considered the gold standard for confirming true immunity and identifying individuals who are susceptible and may need booster doses [12]. However, comprehensive seroprevalence data among HCWs—critical for assessing the effectiveness of hospital vaccination policies in real-world settings—are still limited in many regions.

To address this gap, this study presents a large-scale, cross-sectional evaluation of seroprevalence among HCWs. We conducted a comprehensive serological screening of 1854 individuals working in healthcare facilities across all regions of the country to assess the prevalence of protective IgG antibodies against both measles and rubella. By analyzing this substantial cohort, we aim to map the actual landscape of immunity across different age groups, departments, and professional roles. This analysis will provide data-driven recommendations to strengthen institutional immunization policies and prevent potential hospital outbreaks.

## 2. Materials and Methods

### 2.1. Study Design

The study focused on screening HCWs divided into two main occupational categories: medical doctors (MDs) and non-physician healthcare specialists (referred to hereafter as other medical specialists (MSs), which includes registered nurses, midwives, and laboratory technicians) from all 28 Bulgarian regions. The clinical specimens were collected by Regional Health Inspectorates (RHIs) in connection with the implementation of the National Program for Prevention and Control of Viral Hepatitis in the Republic of Bulgaria, 2021–2025.

### 2.2. Materials

A total of 1854 serum samples collected from HCWs were tested for the presence of IgG antibodies specific for measles and rubella viruses, as an indicator of protective immunity. The laboratory assays were carried out at the National Reference Laboratory “Measles, Mumps, Rubella”, Department of Virology, National Center for Infectious and Parasitic Diseases (NCIPD), Sofia.

### 2.3. Methods

#### Serological Analysis

All serum specimens were tested for the presence of anti-measles and anti-rubella IgG using a commercial indirect enzyme-linked immunosorbent assay (ELISA; Anti-Measles, Anti-Rubella IgG EIA, EUROIMMUN Medizinische Labordiagnostika AG, Lübeck, Germany). The extinction of each tested sample was divided by the extinction of the calibrator, and the results were interpreted qualitatively as positive, negative, or equivocal. In accordance with the manufacturer’s instructions, results were coded as positive when the ratio was ≥1.1, negative when the ratio was <0.8, and borderline when the ratio was 0.8 < but < 1.1, with specificity and sensitivity of more than 95%. Information on independent variables such as gender, age coded in age groups (20–30, 31–40, 41–50, 51–60, 61–70, 71–80, 81+), location (the 28 regions in Bulgaria), profession, and number of years working in the health system was collected and analyzed using statistical methods, which are described below. In our analysis, we consider 1988 (for the female cohort) and 1969 (for all) as potential thresholds for rubella and measles positivity, respectively, as these are years when immunoprophylaxis with monovalent vaccines was introduced.

### 2.4. Statistical Analysis

Bivariate associations were analyzed using Chi-Square tests, with a Monte Carlo simulation for Fisher’s Exact Test employed as an alternative when expected cell counts were low in certain categories. In this way, we corrected for small expected counts and an extensive number of categories when it comes to the location variable. For statistically significant Chi-Square associations, Cramér’s V was calculated to assess the strength of association. Since the outcome variable (serostatus: negative, equivocal, and positive) was inherently ordinal, the Gamma coefficient (γ) was used to assess the strength and direction of associations when evaluating ordinal predictor variables (e.g., age groups).

Multivariable analysis was conducted using ordinal logistic regression to identify independent predictors of higher positivity levels. Duration of employment in healthcare was categorized based on the reported year of entry into healthcare practice, with cut-off points in 2015 and 1994. The extended data collection period (2021–2025) was considered when defining these thresholds, which were selected to ensure approximately balanced group sizes and adequate model stability for multivariable analysis. To account for geographical variation while maintaining model stability, the location variable (comprising 28 districts) was included as a categorical predictor. However, to prevent sparse-data bias, locations with fewer than 20 observations and those with zero cell counts in any of the three antibody outcome categories (“negative”, “equivocal”, or “positive”), most commonly due to zero counts in the equivocal category, were combined into a single group to prevent complete separation and model instability. Adjusted odds ratios (ORs) with 95% confidence intervals (CIs) were calculated, and statistical significance was defined as *p* < 0.05. The HCWs in Plovdiv (*n* = 206) were specified as the reference category for the location variable in the multivariable analysis, as it represented the largest subgroup in the sample. All statistical analyses were performed using IBM SPSS Statistics for Windows, Version 28.0 (IBM Corp., Armonk, NY, USA).

## 3. Results

The study included 1854 HCWs, 1549 females, and 305 males, aged 20 to 82 years (Table 1).

Anti-measles IgG antibodies were detected in 86.1% of participants. The results of 6.4% were equivocal, and 7.44 were negative, suggesting susceptibility to infection. In the case of rubella, 95.2% of health workers were confirmed to have optimal IgG immunity, 1.6% had equivocal immunity, and 3.2% had negative results.

### 3.1. Demographic Factors

The Pearson Chi-Square test did not find any relationships between positivity for measles or rubella and gender (*p* > 0.05).

A statistically significant positive association between measles IgG positivity and age was found (χ^2^(10) = 35.153, *p* < 0.001). The results suggest that samples from older participants were characterized by higher immunity levels (Table 2). However, the strength of the relationship is weak, as indicated by *Cramér’s V* = 0.098 (*p* < 0.001) and the Gamma coefficient (*γ* = 0.208, *p* < 0.001). We also found that participants born before 1969, when the monovalent vaccine for measles was introduced in the population, are more likely to have antibodies for the pathogen than those born after (χ^2^(2) = 23.032, *p* < 0.001, *γ* = 0.372, *p* < 0.001). When analyzed separately by gender, the association between age and positivity remained statistically significant among women (*γ* = 0.207, *p* < 0.001) but was weaker and statistically unstable among men (*γ* = 0.208, Monte Carlo *p* = 0.060). Effect sizes were small in all cases (*Cramér’s V* < 0.10), indicating limited practical significance. Table 2 demonstrates the distribution of results among the age groups.

The data did not show any significant relationship between rubella IgG positivity and age. Although not significantly confirmed and with a minor effect (*Cramér’s V* = 0.009, *p* = 0.939, *γ* = −0.019, *p* = 0.899), we found a relationship indicating that women born before 1988 have lower immunity levels against rubella compared to those born after.

### 3.2. Work-Related Factors

No associations were found between either rubella or measles IgG positivity and the profession reported (*p* > 0.05).

However, rubella IgG positivity appears significantly associated with the hospital ward variable. Our data indicate that staff working in wards different from infectious disease wards (*n+* = 95.7%) are more frequently positive for rubella IgG antibodies compared to staff employed in infectious wards (*n+* = 93.9%), *p* = 0.013. No such significant association was found with measles IgG positivity.

Concerning health sector employment length, no significant associations were found for rubella IgG antibody positivity.

### 3.3. Geographic Distribution

Associations between IgG antibody positivity frequency for both measles and rubella were found with the location of HCWs using a Chi-Square test of independence. The Pearson Chi-Square was significant in both cases:

- Measles: χ^2^_(54, *n* = 1854)_ = 132.772, *p* < 0.001;
- Rubella: χ^2^_(54, *n* = 1854)_ = 173.997, *p* < 0.001.

**Figure 1:**
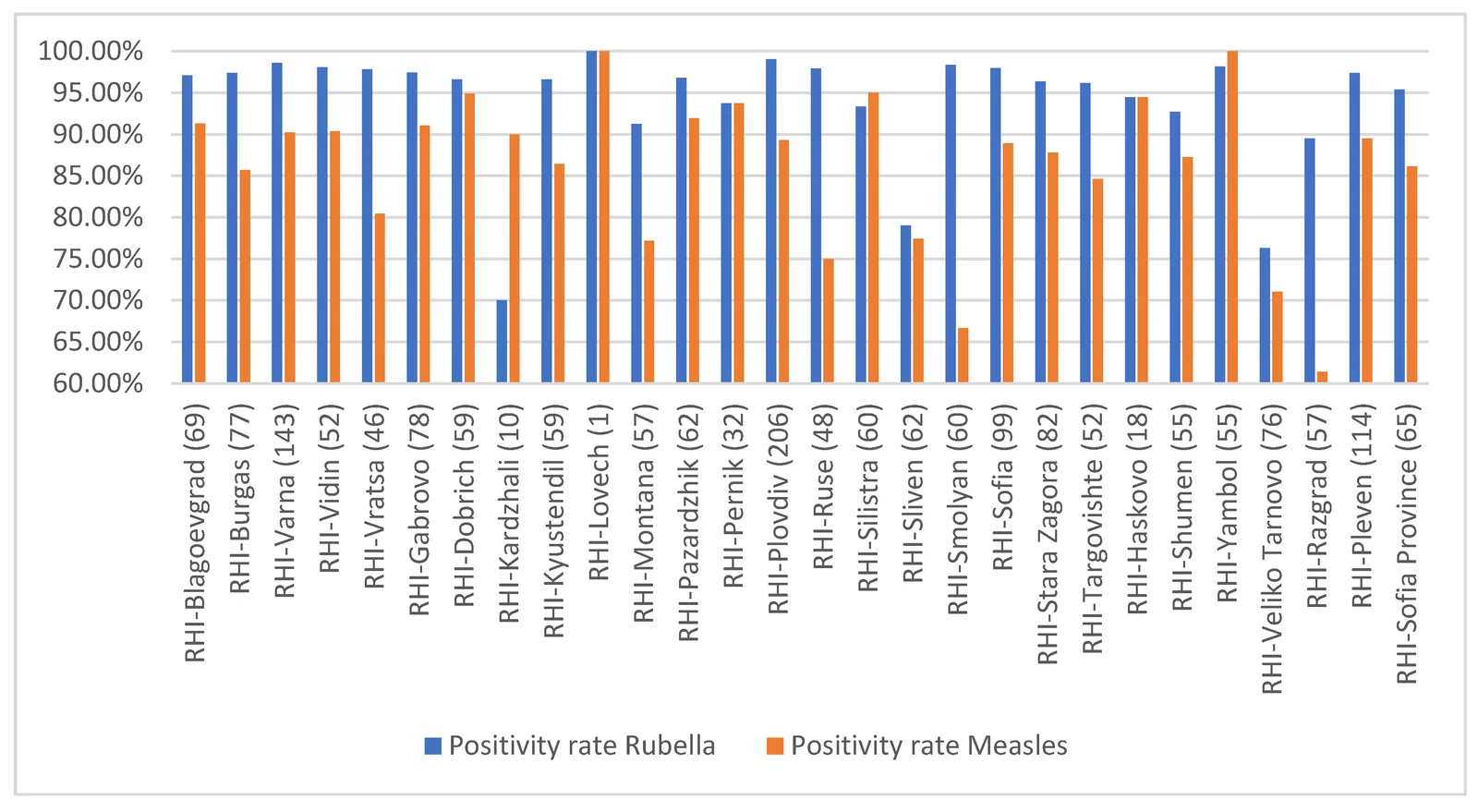
Comparison of measles and rubella IgG seropositivity rates across the 28 Regional Health Inspectorates (RHIs) in Bulgaria. The y-axis starts at 60% to enhance visualization of regional differences; all observed seropositivity rates were greater than 60%. Sample sizes are shown in parentheses next to the region names. Estimates for regions with small sample sizes should be interpreted with caution.

Due to many cells with expected counts less than five, the Monte Carlo simulation method was used to obtain a more accurate *p*-value, which also indicated significance both for rubella and measles (*p* < 0.001). For measles we identified a total IgG positivity of 86.1% with the highest rates (>90%) in the following regions: RHI-Blagoevgrad (91.3%, *n+* = 63), RHI-Varna (90.2%, *n+* = 129), RHI-Vidin (90.4%, *n+* = 47), RHI-Gabrovo (91%, *n+* = 71), RHI-Dobrich (94.9%, *n+* = 56), RHI-Kardzhali (90.0%, *n+* = 9), RHI-Lovech (100%, *n+* = 1), RHI-Pazardzhik (91.9%, *n+* = 57), RHI-Pernik (93.8%, *n+* = 30), RHI-Silistra (95.0%, *n+* = 57), RHI-Haskovo (94.4%, *n+* = 17), Yambol (100%, *n+* = 55). IgG positivity for measles was above 90% in 42.86% of the RHIs in the country (Figure 1).

For rubella, the total IgG positivity of samples reached 95.1%. In nearly all regions (85.71% of all 28 regions), rubella IgG positivity rates exceeded 90%, with a few exceptions: RHI-Veliko Tarnovo (76.3%, *n+* = 58), RHI-Kardzhali (70.0%, *n+* = 7), RHI-Razgrad (89.5%, *n+* = 51), and RHI-Sliven (79.0%, *n+* = 49) (Figure 1).

### 3.4. Multivariable Analysis

#### 3.4.1. Measles

In multivariable ordinal logistic regression, increasing age was independently associated with higher measles IgG positivity (OR = 1.024 per year, 95% CI 1.013–1.036, *p* < 0.001). Gender and professional roles were not significant predictors. Compared with the reference location (RHI-Plovdiv, where positivity level is measured at 89.3%), participants from seven geographic locations had significantly lower odds of measles IgG positivity (ORs 0.179–0.419; Table 3).

#### 3.4.2. Rubella

In multivariable ordinal logistic regression, age, gender, duration of employment and profession were not significantly associated with rubella IgG positivity. Although women had higher odds of positivity compared to men (OR_female_ = 1.44), this association was not statistically significant (95% CI 0.76–2.73, *p* = 0.261). Compared to the reference location RHI-Plovdiv, and controlling for age, gender, and profession, five geographic locations demonstrated significantly lower odds of higher rubella IgG positivity (Table 4).

The magnitude of the association is shown as both regression estimates and odds ratios with 95% confidence intervals.

As previously found by descriptive analyses, the crude rubella IgG seropositivity rate was slightly lower among healthcare workers from infectious disease wards compared with staff from other departments (93.9% vs. 95.7%). To further investigate this difference, an ordinal logistic regression model was performed with rubella IgG serostatus (negative, borderline, or positive) as the ordered outcome. The model included department type, age group, professional category, duration of employment and RHI as explanatory variables. Since the distribution of healthcare personnel among departments differed significantly across RHIs (Pearson χ^2^ = 178.652, df = 24, *p* < 0.001), and regional variation in immunity profiles was considered a potential confounder, RHI was included in the adjusted model. Regions with sparse data were combined to improve model stability. After adjustment, department type was not significantly associated with rubella IgG serostatus (other departments vs. infectious disease wards: β = −0.448, *p* = 0.565).

## 4. Discussion

This study presents a large-scale, nationwide evaluation of measles and rubella seroprevalence among HCWs in Bulgaria, encompassing a substantial cohort of 1854 individuals and providing vital data to assess occupational vulnerability and institutional infection control protocols. It encompasses all 28 districts of Bulgaria, ensuring a highly representative cross-sectional sample of the country’s frontline medical personnel.

The overall seropositivity rate for measles IgG antibodies was 86.1%, indicating that a substantial proportion of the evaluated HCWs have protective immunity. This means that up to 14% of the screened individuals are potentially susceptible to measles infection. This finding is highly concerning when compared to the WHO-recommended herd immunity threshold of 95% required to prevent nosocomial transmission and outbreaks [13].

When compared with the international literature, our measles seropositivity rate (86.1%) is comparable to the pooled European estimate reported among healthcare workers. A recent systematic review and meta-analysis of European HCWs estimated that 13.3% of participants were susceptible to measles, corresponding to an overall seropositivity of approximately 87%, although substantial heterogeneity was observed between countries [9]. Country-specific estimates ranged from lower susceptibility levels in Spain, the Czech Republic, and Hungary to higher proportions of susceptible HCWs in France and Italy [9]. Juxtaposition with earlier seroprevalence research conducted in Italy and France suggests that protection levels have been dramatically decreasing from ~96% [14] to 84.2% [9] and ~90% [15] to 65.8% [9] in the previous decade.

Data from Southeastern Europe remain more limited but indicate persistent immunity gaps among healthcare personnel. Earlier findings from Bulgaria measure anti-measles IgG positivity among HCWs at 85.5% (130/152), closely aligning with results from the fresh data [16]. These findings suggest a stable tendency in the healthcare sector in the country over the last decade. Similarly, a national Bulgarian assessment of HCWs involved specifically in infectious disease care and Regional Health Inspectorates reported measles seropositivity of 82.9%, with 16.6% of participants lacking detectable protective antibodies [17]. In Serbia, among future healthcare workers, only 63.0% demonstrated anti-measles IgG positivity, while 25.6% were seronegative and 11.3% had equivocal results [18]. These findings suggest that immunity gaps among healthcare personnel remain a relevant concern in parts of Southeastern Europe, despite established measles vaccination programmes. Differences in historical vaccine schedules, cohort effects, waning immunity, and incomplete uptake of additional vaccination opportunities may contribute to the observed variability.

The association between age and measles seroprotection observed in our study is in agreement with a European meta-analysis showing increased risk of loss of seroprotection among HCWs born after the introduction of routine measles vaccination [9]. As these cohorts have had limited exposure to circulating wild-type measles virus due to successful immunization programmes, their immunity relies predominantly on vaccine-induced protection. This may partly explain the lower seropositivity observed among younger HCWs and highlights the importance of monitoring immunity in post-vaccination generations. The antibody profile observed in the middle-aged cohort (41–60 years) warrants careful interpretation regarding the mechanisms of vaccine-induced versus natural immunity. In Bulgaria, monovalent measles vaccination was introduced in 1969. Consequently, individuals in the 41–60 age group belong to a transitional generation exposed to both early monovalent live-attenuated vaccine regimens and ongoing natural viral circulation during childhood outbreaks. The proportion of non-protective or borderline antibody levels in this subgroup may stem from two distinct mechanisms: primary vaccine failure (e.g., lack of initial seroconversion due to historical cold-chain/storage limitations or host immune non-responsiveness to the early live vaccine strains) and secondary vaccine failure (the gradual waning of vaccine-induced antibody titers over several decades in the absence of natural wild booster exposures). Unlike individuals over 60 years old who overwhelmingly possess robust, lifelong humoral immunity from natural childhood infection, vaccines in the 41–60 age bracket are more susceptible to waning IgG levels over time, underscoring the necessity of periodic serological assessment and booster recommendations for HCWs.

In contrast, our cohort demonstrated a significantly higher level of protection against rubella, with 95.2% of HCWs showing optimal IgG immunity and data indicating that only around 5% of HCWs remain unprotected. While this level of protection is encouraging, it still requires a targeted strategy and approach when attending to immunocompromised patients to minimize risk. The presence of non-immune HCWs within ob-gyn healthcare structures presents a critical risk for nosocomial transmission, particularly to pregnant patients, as it significantly increases the risk of CRS [19,20]. The slightly lower crude rubella IgG seropositivity observed among healthcare workers from infectious disease wards compared with other departments was unexpected, as greater occupational exposure might theoretically be associated with higher immunity. However, this difference was not maintained after adjustment for potential confounding factors, including age group, professional category, duration of employment and geographic region. The lack of an independent association between department type and rubella IgG serostatus suggests that occupational placement alone does not explain differences in rubella immunity among healthcare workers.

The significant regional variation observed in the adjusted analysis highlights the importance of geographic factors in shaping immunity profiles. Differences in historical vaccination coverage, previous circulation of rubella virus, population immunity, and healthcare workforce composition may contribute to regional variation. In Bulgaria, the rubella vaccination strategy commenced in 1988 and focused on selective immunization with a monovalent vaccine, primarily targeting girls to prevent cases of CRS. The wild virus spread easily among non-immune populations, and the milder nature of the disease contributed to a greater overall immunity within the population. However, in our current data, information on individual MMR vaccination history, booster doses, and occupational protective behaviours was not available and could not be assessed.

Our study findings reveal the age-specific distribution of measles IgG immunity and higher measles protection in older HCWs (51–60 years old). The older generations (typically over 50 years) usually have robust, lifelong immunity due to prior childhood exposure to wild-type viruses [18,19]. However, we did not find any significant relationship between rubella IgG immunity and age.

We observed lower seropositivity rates for measles in the 20–30-year-old age group. This cohort represents a primary “immunity gap” in Bulgarian HCWs, highlighting the urgent need for targeted screening and intervention. Individuals in this age range were born during a time when, in Bulgaria, the standardized two-dose MMR vaccination regimens were introduced. Natural viral circulation was declining due to vaccination campaigns; however, vaccine formulation, dosing, and coverage had not yet been fully optimized [19].

We found that positivity for rubella IgG was significantly associated with the hospital ward variable related to work and a higher immunity threshold for those working in non-infectious wards. Such findings were surprising given the recently registered measles outbreaks in 2017 and 2019 managed by wards specialized in infections.

Although the overall seropositivity observed in the present study is comparable to the pooled European estimate for healthcare workers, approximately one in seven HCWs remained susceptible to measles. Because measles is one of the most contagious human infections (R_0_ typically estimated at 12–18), maintaining ≥95% immunity is considered necessary to interrupt transmission. Consequently, an overall seropositivity of 86.1% indicates that susceptible HCWs may facilitate nosocomial transmission, particularly in departments caring for immunocompromised patients, infants, pregnant women, and individuals who cannot be vaccinated. Previous European hospital outbreaks have demonstrated that unvaccinated HCWs can both acquire and transmit measles, resulting in secondary cases among patients and healthcare personnel [9,21].

Regarding professional roles, while our multivariable model did not show a statistically significant difference in seropositivity between MDs and other MSs, it is important to note that nurses and frontline allied healthcare personnel often experience higher direct patient contact and longer duration of potential exposure than physicians. Clear occupational classification allows for better identification of high-risk operational units within hospitals. The substantial proportion of susceptible doctors and medical specialists in many of the 28 districts emphasizes that relying strictly on self-reported history or historical vaccine documentation is an unreliable strategy for occupational health [10]. Our results advocate for the implementation of routine pre-employment serological screening. Our findings support the implementation of structured measles immunity assessments among healthcare workers, with prevention strategies tailored according to occupational risk. Pre-employment verification of measles immunity and MMR vaccination of susceptible personnel should be prioritized, particularly among HCWs working in high-risk clinical areas, including paediatrics, neonatology, infectious diseases, obstetrics, emergency departments, and units caring for immunocompromised patients. For these departments, documented evidence of immunity or completion of an appropriate vaccination schedule should be ensured before direct patient contact whenever feasible. Non-immune or equivocal personnel should be promptly offered the MMR vaccine to close these institutional immunity gaps, especially in high-risk units like pediatrics, obstetrics, and infectious diseases.

In addition, prevention strategies should consider differences between HCW population groups and geographic regions. Younger personnel, individuals without documented vaccination history, and HCWs from areas with lower observed seropositivity may represent priority groups for targeted assessment and intervention. Region-specific surveillance of HCW immunity could support more efficient allocation of vaccination and monitoring resources. Together, these measures may reduce the risk of healthcare-associated measles transmission and improve preparedness for imported measles cases.

## 5. Strengths and Limitations

One of the major strengths of this study is its large sample size of 1854 individuals and its comprehensive coverage across all 28 administrative districts of Bulgaria. By using standardized, high-quality commercial ELISA assays, we ensured that our findings are both reproducible and clinically relevant.

However, this study has some limitations. Its cross-sectional design precludes the assessment of changes in antibody titers over time or establishment of causal relationships between occupational characteristics and serological status. While department type, including work in infectious disease wards, was considered as a proxy indicator of occupational exposure intensity and frequency of contact with infectious patients, it does not capture the full variability of individual exposure patterns, including specific patient contacts, use of protective measures, and duration or frequency of exposure events. Additionally, we did not have complete access to the immunization records (including application of booster doses) of all participants, which restricts our ability to directly correlate the number of vaccine doses received with current antibody levels. Information on previous clinical history of measles or rubella infection was also unavailable, which may have resulted in residual confounding. Despite these limitations, our data provide a valuable and objective snapshot of real-world immunity within the Bulgarian healthcare system.

## 6. Conclusions

HCWs are at a greater risk of contracting infections than the general public because they have contact with sick patients and infectious material. Infected healthcare workers can spread nosocomial diseases to vulnerable patients with more severe illness, complications, and even death. Therefore, the vaccination and IgG immunity status of HCWs must be strictly monitored to limit the spread of nosocomial infections in hospital settings and should be required when entering work, especially in infectious disease wards.

## Figures and Tables

**Table 1:** Demographic and occupational profile of the study participants. (*n* = 1854).

Characteristics		*n*	(%)
**HCWs Tested**		1854	(100)
Gender	Female	1549	(83.6)
	Male	305	(16.4)
Occupation	MDs	541	(29.2)
	MS	1313	(70.8)

**Table 2:** Heatmap showing the distribution of measles IgG serostatus within age groups. Each cell displays the number and percentage of IgG-negative, borderline, and IgG-positive participants within the respective age group. Colour intensity reflects the proportion within each age group: red for IgG-negative; yellow for borderline; yellow-to-green for IgG-positive; and darker shades indicating higher proportions.

Anti-Measles IgG Results	Age Groups *n* (%)					
	20–30	31–40	41–50	51–60	61–70	71+
negative	21 (7.9%)	25 (8.7%)	41 (10.1%)	39 (7.4%)	11 (3.5%)	1 (2.5%)
borderline	21 (7.9%)	23 (8.0%)	37 (9.1%)	31 (5.9%)	7 (2.2%)	0 (0.0%)
positive	224 (84.2%)	239 (83.3%)	329 (80.8%)	458 (86.7%)	298 (94.3%)	39 (97.5%)

**Table 3:** Locations with significantly lower odds of higher measles IgG positivity compared to the reference (RHI-Plovdiv, *n* = 206, positivity 89.3%). The magnitude of the association is shown as both regression estimates and odds ratios with 95% confidence intervals.

Location	Estimate (β)	Estimate 95% CI	OR	OR 95% CI	*p*-Value
Vratsa	−0.87	−1.709–−0.031	0.419	0.181–0.969	0.042
Montana	−0.887	−1.659–−0.115	0.412	0.190–0.891	0.024
Ruse	−1.027	−1.815–−0.239	0.358	0.163–0.787	0.011
Sliven	−1.097	−1.839–−0.355	0.334	0.159–0.701	0.004
Smolyan	−1.487	−2.181–−0.793	0.226	0.113–0.452	<0.001
Veliko Tarnovo	−1.214	−1.892–−0.535	0.297	0.151–0.586	<0.001
Razgrad	−1.724	−2.417–−1.031	0.179	0.089–0.356	<0.001

**Table 4:** Locations with significantly lower odds of higher rubella positivity compared to the reference (RHI-Plovdiv, *n* = 206, positivity 89.3%).

Location	Estimate (β)	Estimate 95% CI	OR	OR 95% CI	*p*-Value
Montana	−2.318	−4.000–−0.636	0.10	0.024–0.761	0.007
Silistra	−2.004	−3.734–−0.273	0.14	0.008–0.173	0.023
Sliven	−3.293	−4.828–−1.759	0.04	0.007–0.135	<0.001
Veliko Tarnovo	−3.507	−5.010–−2.004	0.03	0.016–0.406	<0.001
Razgrad	−2.537	−4.172–−0.902	0.08	0.024–0.761	0.002

## Data Availability

Data is unavailable due to privacy or ethical restrictions.

## Abbreviations

The following abbreviations are used in this manuscript:

HCWs	Healthcare workers
MDs	Medical doctors
MS	Medical specialists
WHO	World Health Organization
VPDs	Vaccine-preventable diseases
CRS	Congenital rubella syndrome
ELISA	Enzyme-linked immunosorbent assay
RHIs	Regional health inspectorates
IgG	Immunoglobulin G
